# Supplementary material for: Subject-specific body segment parameter estimation using 3D photogrammetry with multiple cameras
Source: PeerJ. 2015 Mar 10;3:e831. doi: 10.7717/peerj.831 (PMC4359122; doi:10.7717/peerj.831)
Supplement: Supplemental Information [file peerj-03-831-s001.pdf]

Supporting Information

Subject-specific body segment parameter estimation using 3D photogrammtery with multiple cameras

Kathrin Eva Peyer, Mark Morris, William Irvin Sellers

S1. Methods

S1.1. Photogrammetric reconstruction

The photogrammetric reconstruction was performed in Agisoft PhotoScan Standard Edition (http://www.agisoft.com). The *Accuracy* parameter during the photo-alignment step was set to “high” and *Pair preselection* was disabled. For the dense point cloud reconstruction, the *Quality* parameter was set to “high”, and the *Depth Filtering Mode* to “mild”.

Before switching to AgiSoft Photoscan, the open-source programme VisualSFM was used for the photogrammetric reconstruction. VisualSFM gives access to more parameters of the reconstruction algorithm, which made it more difficult to find an optimal set of parameters. For the readers interested in using VisualSFM, the following parameter description (based on the documentation provided here on http://www.di.ens.fr/pmvs/documentation.html) may be useful.

VisualSFM parameters description:

Level: Default: 1. Image sub-sampling level. A value of 0 makes use of the full resolution image. This leads to an increase in computation time and (interestingly) does not always improve the result (because the sub-sampling can have a noise-reducing effect on the images). Increasing the value decreases the computation time, but will cause feature loss.

Csize: Default: 2. Density of patch reconstruction. Decreasing csize (to a minimal value of 1) allows for more dense point cloud reconstruction (at the cost of more computation time).

Threshold: Default: 0.7. Patch matching acceptance criterion. By decreasing this value more points are reconstructed, however at the cost of including false points. Increasing the value causes loss of data points.

Wsize: Default: 7. Wsize x Wsize pixels corresponds to the size of the patch used for matching. Increasing this value helped with less noisy reconstructions in some trials, but in many others did not significantly affect the results, and the default appears to be a good choice.

MinNumIm: Default: 3. Minimum number of images in which one feature is detected to estimate its location in 3D (value cannot be smaller than 2). Should be chosen smaller than or equal to number of images containing the same feature point (if a too large number of images is chosen, the feature point will not be recognised). Decreasing this value allowed a more dense reconstruction, but at the cost of less precise 3D position estimations, causing smooth surfaces to appear rugged.

MaxAngle: Default: 10. Minimal angle (in degrees) between the lines from a (reconstruction) point to two adjacent cameras (containing that point) that still gets reconstructed. Value should be chosen just below the smallest angle found in the setup. The smallest angle is given by the feature point furthest away from two adjacent cameras. Decreasing this value further includes noise in the background, whereas increasing leads to loss of features

CPU: Default: 0.The number of cores used for running the algorithm. Default 0 uses actual number of cores. The algorithm can use “virtual” cores, and a value of double the number of the actual number of cores is recommendable to decrease computation time.

S1.2 Convex hull computation

The convex hull algorithm is readily available in a number of software packages and as a library on www.qhull.org. In fact, both Matlab and Meshlab utilise the Qhull library. In Matlab (the approach used in this paper), the convex hull can be computed using the *convhull* command. In Meshlab, the convex hull command can be found under *Filters* > *Remeshing, Simplification, Reconstruction* > *Convex Hull*.

S1.3 Segment mass and inertial tensor computation

Based on the convex hull mesh, the mass and inertial tensor values are computed numerically. In this paper, a Matlab script was implemented to calculate the inertial parameters of a volume described by a surface mesh (see below). Alternativly, Meshlab provides a function to computes the volume and inertial tensor (amongst other information),

which can be found under *Filters > Quality Measure and Computations > Compute Geometric Measures*. To see the output displayed, the Layer Dialogue window has to activated under *View > Show Layer Dialogue*. Because the display is limited to 8 digits, it is advisable to work in centimetre units (instead of metre or millimetre) as the inertial tensor values would otherwise not be displayed well. The scaling of the model can also be done in Meshlab using *Filters > Normals, Curvature and Orientations > Scale*.

The numerical computation of the volume (of segment i) $V_{hull,i}$ has to be multiplied by the body segment densities ρ_i to get the mass of the convex hulls $m_{hull,i}$.

$$m_{hull,i} = V_{hull,i} \cdot \rho_i \quad \text{(S1)}$$

The computed total body mass (i.e. sum of all body hull masses) was never an exact match to the subject body mass measured. A pro-rata scaling factor s was thus used to adjust the body densities and thus final body segment mass $m_{segm,i}$ estimation.

$$s = \frac{M_{subject}}{\sum m_{hull,i}} \quad \text{(S2)}$$

$$m_{segm,i} = m_{hull,i} \cdot s \quad \text{(S3)}$$

The scaling factor effectively scales the body densities, and was thus applied to the mass as well as the inertial tensor values.

The numerical computations of the inertial tensor (in both Matlab or Meshlab) assume a density of 1, thus the values have to be multiplied by the body segment densities (which are adjusted using the scaling factor).

$$I_{segm,i} = I_{hull,i} \cdot \rho_i \cdot s \quad \text{(S4)}$$

Matlab script to compute volume and inertial tensor values of a closed mesh surface:

Pseudo code derived by David Eberly, reported in "Polyhedral Mass Properties (Revisited)", available at: http://geometrictools.com/Documentation/PolyhedralMassProperties.pdf. Translated into Matlab code by Kathrin E. Peyer.

```
function [mass,cm,inertia] = ComputeBodyProperties(p,index)


mult = [1/6,1/24,1/24,1/24,1/60,1/60,1/60,1/120,1/120,1/120];

intg = [0,0,0,0,0,0,0,0,0,0];    % order: 1, x, y, z, x^2, y^2, z^2, xy, yz,
zx
```

```
for t = 1:length(index)

    % get vertices of triangle t
    i0 = index(t,1);
    i1 = index(t,2);
    i2 = index(t,3);

    x0 = p(i0,1);
    y0 = p(i0,2);
    z0 = p(i0,3);

    x1 = p(i1,1);
    y1 = p(i1,2);
    z1 = p(i1,3);

    x2 = p(i2,1);
    y2 = p(i2,2);
    z2 = p(i2,3);


    % get edges and cross product of edges
    a1 = x1-x0;
    b1 = y1-y0;
    c1 = z1-z0;
    a2 = x2-x0;
    b2 = y2-y0;
    c2 = z2-z0;
    d0 = b1*c2-b2*c1;
    d1 = a2*c1-a1*c2;
    d2 = a1*b2-a2*b1;


    % compute integral terms
    [f1x,f2x,f3x,g0x,g1x,g2x] = Subexpressions(x0,x1,x2);
    [f1y,f2y,f3y,g0y,g1y,g2y] = Subexpressions(y0,y1,y2);
    [f1z,f2z,f3z,g0z,g1z,g2z] = Subexpressions(z0,z1,z2);


    % update integrals
    intg(1) = intg(1) + d0*f1x;
    intg(2) = intg(2) + d0*f2x;
    intg(3) = intg(3) + d1*f2y;
    intg(4) = intg(4) + d2*f2z;
    intg(5) = intg(5) + d0*f3x;
    intg(6) = intg(6) + d1*f3y;
    intg(7) = intg(7) + d2*f3z;
    intg(8) = intg(8) + d0*(y0*g0x+y1*g1x+y2*g2x);
    intg(9) = intg(9) + d1*(z0*g0y+z1*g1y+z2*g2y);
    intg(10) = intg(10) + d2*(x0*g0z+x1*g1z+x2*g2z);
end

intg = intg.*mult;

mass = intg(1);

%% Center of mass:
% CoM.x = cm(1)
```

```matlab
% CoM.y = cm(2)
% CoM.z = cm(3)

cm(1) = intg(2)/mass;
cm(2) = intg(3)/mass;
cm(3) = intg(4)/mass;

%% Inertia: given wrt center of mass
% I11  = inertia(1)
% I22  = inertia(2)
% I33  = inertia(3)
% I12  = inertia(4)
% I13  = inertia(5)
% I23  = inertia(6)

inertia(1) = intg(6)+intg(7)-mass*(cm(2)*cm(2)+cm(3)*cm(3));
inertia(2) = intg(5)+intg(7)-mass*(cm(3)*cm(3)+cm(1)*cm(1));
inertia(3) = intg(5)+intg(6)-mass*(cm(1)*cm(1)+cm(2)*cm(2));
inertia(4) = -(intg(8)-mass*cm(1)*cm(2));
inertia(5) = -(intg(10)-mass*cm(3)*cm(1));
inertia(6) = -(intg(9)-mass*cm(2)*cm(3));

end


function [f1,f2,f3,g0,g1,g2] = Subexpressions(w0,w1,w2)
    temp0 = w0+w1;
    temp1 = w0*w0;
    temp2 = temp1+w1*temp0;

    f1 = temp0+w2;
    f2 = temp2+w2*f1;
    f3 = w0*temp1+w1*temp2+w2*f2;
    g0 = f2+w0*(f1+w0);
    g1 = f2+w1*(f1+w1);
    g2 = f2+w2*(f1+w2);
end
```

S1.4 Segment definition

Table S1: Reference points (R1 and R2) for calculating segment lengths and relative CoM. CoM percentage given as distance from the reference point indicated with an * with respect to the segment length.

Segment	R1	R1 Description	R2	R2 Description
Foot	TTIP*	Acropodion – tip of longest toe	HEEL	Pternion - Posterior point on heel
Shank	LMAL	Most lateral point on lateral malleolus	KJC*	Knee joint centre
Thigh	KJC	Knee joint centre	ILIO*	Anterior Illiospinale
Hand	DAC3*	Tip of the 3rd digit	WJC	Wrist joint centre
Forearm	WJC*	Wrist joint centre	ULNA	Olecranon
Upper Arm	OLEC*	Olecranon	ACR	Acromonion
Head and Neck	CERV	Cervicale (C7)	VERT*	Vertex
Torso	HSP int	Intersection of hip segmentation planes	CERV*	Cervicale (C7)

S2. RPi Scanner Data

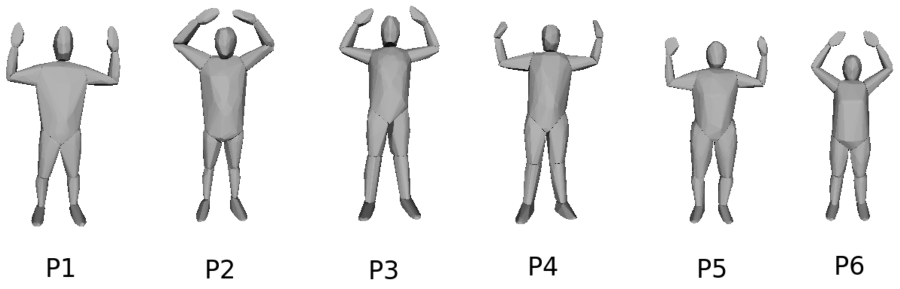

Figure S1: Segmented convex hull of participants P1 – P6.

S2.2. Raw data of mass, centre of mass, segmental lengths, moment and product of inertia

Table S2: Product of inertia Ixy in [10^4 kg*m²]. The definition of the coordinate system is shown in Fig. 2. Products of inertia sign convention such that appears as -Ixy in the inertia matrix.

Segment	P1 (m)	P2 (m)	P3 (m)	P4 (m)	P5 (f)	P6 (f)
Head and Neck	0.45	-16.53	4.56	4.60	-0.79	-1.04
Left Arm	1.08	0.31	-3.21	-0.51	0.56	-0.91
Left Foot	-2.61	-0.10	-0.39	-0.90	-0.29	-0.43
Left Forearm	-0.51	0.07	-0.71	-0.03	0.07	-0.26
Left Hand	0.07	0.41	-0.21	0.26	0.12	-0.19
Left Shank	1.91	0.34	-6.33	1.31	2.07	-1.11
Left Thigh	-0.69	7.60	74.02	21.09	27.37	15.43
Right Arm	0.33	4.44	2.92	-0.10	1.23	0.47
Right Foot	-1.12	0.59	1.64	2.39	1.62	-0.04
Right Forearm	0.47	1.58	1.60	0.47	0.09	0.20
Right Hand	-0.11	-0.24	0.12	0.04	0.03	-0.03
Right Shank	-2.56	-0.10	3.25	2.22	-1.37	-0.83
Right Thigh	4.79	-5.59	-22.95	-3.02	-26.27	-29.82
Torso	-117.47	6.63	66.85	-176.34	50.21	17.93

Table S3: Product of inertia Ixz in [10^4 kg*m²]. The definition of the coordinate system is shown in Fig. 2. Products of inertia sign convention such that appears as -Ixz in the inertia matrix.

Segment	P1 (m)	P2 (m)	P3 (m)	P4 (m)	P5 (f)	P6 (f)
Head and Neck	-8.45	-3.42	14.33	-8.72	-8.22	-2.55
Left Arm	-5.60	-0.27	1.30	0.30	-1.89	0.68
Left Foot	4.28	3.75	8.35	3.53	2.21	2.61
Left Forearm	-0.96	-3.24	-0.01	-1.57	0.27	0.16
Left Hand	-0.53	-0.21	0.72	1.68	-0.10	0.30
Left Shank	15.95	12.14	69.01	13.04	19.62	12.69
Left Thigh	174.73	138.77	194.88	43.90	119.96	47.81
Right Arm	-6.32	-7.94	2.95	5.16	-1.66	-1.72
Right Foot	3.02	3.65	3.62	7.58	2.33	1.67
Right Forearm	-0.88	1.72	0.69	-0.52	0.19	-0.32
Right Hand	-0.01	-0.30	-0.08	-0.11	0.09	-0.04
Right Shank	23.95	3.53	57.18	49.98	26.96	9.40
Right Thigh	137.64	125.26	128.32	18.07	100.20	36.73
Torso	-940.60	-1741.76	-1781.19	-606.51	-185.74	-185.17

Table S4: Product of inertia Iyz in [10^4 kg*m²]. The definition of the coordinate system is shown in Fig. 2. Products of inertia sign convention such that appears as -Iyz in the inertia matrix.

Segment	P1 (m)	P2 (m)	P3 (m)	P4 (m)	P5 (f)	P6 (f)
Head and Neck	-1.99	-2.95	2.86	-0.27	-1.90	0.29
Left Arm	10.03	4.73	8.21	10.37	2.87	-1.50
Left Foot	0.77	0.78	0.80	-0.34	0.08	0.08
Left Forearm	-0.92	-4.36	-1.56	-1.10	-0.05	0.23
Left Hand	-0.12	-0.86	-1.49	1.32	-0.14	-0.19
Left Shank	9.70	8.24	-34.25	-8.89	37.97	7.31
Left Thigh	37.59	110.50	121.73	232.66	136.73	92.79
Right Arm	-17.19	-4.22	-5.41	-13.21	-7.09	-0.47
Right Foot	-0.30	-1.14	-0.45	-0.47	-0.37	-0.33
Right Forearm	1.70	4.29	-0.11	-0.35	0.06	0.23
Right Hand	0.05	0.49	0.42	-0.17	0.06	0.44
Right Shank	-13.56	-0.52	24.28	30.34	-42.75	-0.75
Right Thigh	4.29	-63.23	-81.10	-166.71	-179.01	-82.07
Torso	140.90	-149.09	343.34	951.10	173.17	-7.95

Table S5: Moment of inertia Ixx [1e4 * kg*m²]. The definition of the coordinate system is shown in Fig. 2.

Segment	P1 (m)	P2 (m)	P3 (m)	P4 (m)	P5 (f)	P6 (f)	Z (m)	Z (f)
Foot	10.7	15.0	13.8	16.7	7.6	7.8	10.3	8.9
Shank	263.2	352.8	904.7	708.7	360.1	271.7	385.0	409.9
Thigh	1549.4	1057.7	1407.2	1637.8	1805.6	862.4	1997.8	1690.1
Hand	7.9	12.2	17.4	11.4	5.4	4.2	13.2	6.0
Forearm	34.1	57.6	48.0	26.5	20.9	19.8	64.2	40.9
Upper Arm	127.1	178.5	187.9	181.0	69.2	88.5	127.3	92.3
Head and Neck	253.3	327.9	261.5	238.1	140.6	139.2	272.1	183.6
Torso	15696.4	19239.4	18431.1	21112.2	8832.6	8433.8	12421.1	9408.8

Table S6: Moment of inertia Iyy [1e4 * kg*m²]. The definition of the coordinate system is shown in Fig. 2.

Segment	P1 (m)	P2 (m)	P3 (m)	P4 (m)	P5 (f)	P6 (f)	Z (m)	Z (f)
Foot	41.3	43.9	53.2	54.1	22.9	22.8	44.0	41.1
Shank	266.3	354.8	923.6	727.1	356.6	274.6	371.0	399.7
Thigh	1624.4	1108.8	1513.6	1724.9	1887.1	904.5	1999.4	1647.3
Hand	6.6	11.1	15.0	9.5	4.2	3.5	8.8	4.4
Forearm	33.6	57.1	47.9	25.7	20.5	19.5	60.2	39.7
Upper Arm	116.8	172.9	189.5	170.7	64.9	85.6	114.4	80.7
Head and Neck	307.6	380.5	306.5	287.7	166.7	162.9	293.9	216.5
Torso	13500.9	17694.8	17569.8	19587.2	7897.9	7913.6	10807.7	8483.9

Table S7: Moment of inertia Izz [1e4 * kg*m²]. The definition of the coordinate system is shown in Fig. 2.

Segment	P1 (m)	P2 (m)	P3 (m)	P4 (m)	P5 (f)	P6 (f)	Z (m)	Z (f)
Foot	42.4	45.2	54.8	53.4	22.5	23.7	40.0	35.6
Shank	50.0	44.0	148.9	143.4	74.8	59.5	64.6	48.6
Thigh	475.8	297.2	578.1	641.0	609.6	332.3	413.4	324.2
Hand	2.0	3.2	5.1	3.7	1.8	1.2	5.4	2.4
Forearm	5.7	10.1	9.3	5.4	3.2	2.6	12.6	5.3
Upper Arm	42.0	46.5	57.0	57.3	24.1	18.2	39.0	26.2
Head and Neck	187.0	253.3	190.9	191.1	125.6	113.2	202.4	172.7
Torso	5518.1	6327.0	7339.2	7104.4	3642.1	3124.6	3274.5	2158.7

Table S8: Segment lengths [m] (average between left and right). The definition of the segments and reference points is given in Table A1 - Exceptions: * Foot of P1 and P2: Heel and toe end point of participant's shoes instead of foot. ** Forearm and Upper Arm of Z: Elbow reference point is the elbow joint centre instead of the Olecranon.

Segment	P1 (m)	P2 (m)	P3 (m)	P4 (m)	P5 (f)	P6 (f)
Foot	0.311	0.299	0.319	0.307	0.260	0.261
Shank	0.358	0.405	0.463	0.414	0.375	0.342
Thigh	0.512	0.506	0.467	0.501	0.530	0.433
Hand	0.197	0.209	0.221	0.198	0.165	0.165
Forearm	0.275	0.296	0.284	0.249	0.261	0.247
UpperArm	0.329	0.338	0.330	0.346	0.282	0.321
HeadAndNeck	0.263	0.279	0.255	0.259	0.219	0.226
Torso	0.732	0.800	0.741	0.802	0.649	0.656

Table S9: Segment mass (as % of body weight). P1 - P6: Participants. Z(m): Male average values reported by Zatsiorsky. Z(f): Female average values reported by Zatsiorsky (Leva, 1996; Zatsiorsky, 2002). D(m): Male average values by Dempster (via Zatsiorsky) (Dempster, 1955; Zatsiorsky, 2002).

**Segment**	**P1 (m)**	**P2 (m)**	**P3 (m)**	**P4 (m)**	**P5 (f)**	**P6 (f)**	**Z (m)**	**Z (f)**	**D (m)**
Foot	1.01	1.13	0.97	1.04	0.87	1.04	1.37	1.29	1.44
Shank	3.71	3.66	6.04	5.63	5.05	5.29	4.33	4.81	4.63
Thigh	12.62	9.14	10.84	11.60	16.20	12.62	14.17	14.78	9.93
Hand	0.45	0.60	0.65	0.49	0.44	0.40	0.61	0.56	0.62
Forearm	1.03	1.36	1.09	0.77	0.83	0.88	1.63	1.38	1.59
Upper Arm	2.78	3.12	2.92	2.80	2.23	2.55	2.7	2.55	2.73
Head and Neck	6.09	6.90	5.04	4.90	5.07	5.71	6.94	6.68	8.13
Torso	50.73	55.07	49.94	50.44	43.69	48.72	43.47	42.57	50.00

Table S10: Centre of mass along the longitudinal axis. P1 - P6: Participants. Z(m: male, f: female): Average values by Zatsiorsky, adjusted by de Leva . The CoM is given as % of the segment length. The definition of the segments and reference points are given in Table S1 - Exceptions: * Foot of participants: Heel and toe end point of participant's shoes instead of foot. ** Forearm and Upper Arm of Z: Elbow reference point is the elbow joint centre instead of the Olecranon (Leva, 1996; Zatsiorsky, 2002).

**Segment**	**P1 (m)**	**P2 (m)**	**P3 (m)**	**P4 (m)**	**P5 (f)**	**P6 (f)**	**Z (m)**	**Z (f)**
Foot	61.1	56.4	57.7	56.9	57.8	59.2	55.9	59.9
Shank	44.9	47.6	45.8	46.1	44.8	47.0	40.5	40.3
Thigh	48.4	50.6	51.8	52.3	50.3	51.3	45.5	46.1
Hand	40.5	58.5	57.1	54.2	53.0	41.2	63.1	65.0
Forearm	47.7	51.7	52.7	53.3	54.4	44.8	57.3	57.4
Upper Arm	42.0	59.3	61.2	58.4	56.8	41.5	55.0	56.0
Head and Neck	50.0	49.1	52.7	50.7	51.9	53.7	50.0	48.4
Torso	49.3	46.5	47.1	47.7	47.6	50.3	43.7	44.2

Table S11: CoM shift in x-direction from the anatomical longitudinal axis in the transverse (x-y) plane. The CoM is given as % of the segment length. The data of the foot is not included due to the participants wearing shoes.

**Segment**	**P1**	**P2**	**P3**	**P4**	**P5**	**P6**
Head	-0.13	2.71	-1.24	1.07	0.79	1.78
Left Arm	0.27	-0.23	-0.40	-0.35	0.54	-0.43
Left Forearm	0.78	0.39	-0.08	0.22	-0.17	-0.18
Left Hand	0.83	0.64	-0.27	-0.20	0.17	-0.20
Left Shank	-1.08	0.13	0.80	-0.73	-1.59	-1.18
Left Thigh	-1.19	-1.66	-2.64	-0.29	-0.40	0.19
Right Arm	0.52	0.58	-0.58	-0.34	-1.38	-0.26
Right Forearm	0.37	0.07	-0.05	-0.02	0.29	0.33
Right Hand	0.19	0.64	0.42	0.03	0.14	0.13
Right Shank	-2.64	0.21	-2.76	-2.79	-1.72	-1.85
Right Thigh	-0.66	-1.29	0.05	0.61	-0.49	0.01
Torso	0.35	1.50	1.81	1.14	1.24	2.59

Table S12: CoM shift in y-direction from the anatomical longitudinal axis in the transverse (x-y) plane. The CoM is given as % of the segment length. The data of the foot is not included due to the participants wearing shoes.

**Segment**	**P1**	**P2**	**P3**	**P4**	**P5**	**P6**
Head	-0.16	-0.39	-0.17	-0.34	-0.30	-0.13
Left Arm	-0.56	-0.13	-0.43	-0.89	0.00	0.11
Left Forearm	-0.74	0.43	-0.93	-0.52	-0.43	-0.62
Left Hand	1.05	0.37	0.75	0.04	0.38	-0.05
Left Shank	-0.01	1.04	1.15	0.43	-0.10	1.14
Left Thigh	1.00	0.79	0.46	0.59	1.08	0.30
Right Arm	-0.02	-0.26	0.18	0.16	1.40	0.40
Right Forearm	0.28	0.02	0.34	0.78	0.62	0.43
Right Hand	-0.33	-0.66	-0.22	0.26	-0.05	-1.22
Right Shank	-0.05	-0.34	-0.73	-1.06	0.77	-0.74
Right Thigh	-0.56	-0.85	-0.26	0.00	-1.27	0.28
Torso	-1.16	0.39	-0.62	-1.63	-0.33	-0.11

Table S13: Segment lengths as % of body height (average between left and right). The definition of the segments and reference points is given in Table A1 - Exceptions: * Foot: Heel and toe end point of participant's shoes instead of foot.

**Segment**	**P1 (m)**	**P2 (m)**	**P3 (m)**	**P4 (m)**	**P5 (f)**	**P6 (f)**
Foot	17.192	16.377	17.208	16.795	15.783	16.478
Shank	19.822	22.178	25.032	22.708	22.784	21.637
Thigh	28.328	27.707	25.232	27.444	32.235	27.369
Hand	10.884	11.451	11.942	10.840	10.043	10.446
Forearm	15.234	16.217	15.357	13.627	15.836	15.630
Upper Arm	18.186	18.513	17.827	18.971	17.164	20.276
Head and Neck	14.541	15.273	13.801	14.212	13.302	14.289
Torso	40.475	43.821	40.013	43.951	39.457	41.479

S3. Visible Human Data

Table S14: Volume of body segments in [1e3*m³]. S: Original surface mesh. CH: Convex hull of body segment. CHD: Convex hull of divided body segments (only segments indicated with an * were subdivided).

Segment	S	CH	CHD
Head	5.72	6.06	N/A
Left Arm	3.20	3.39	N/A
Left Foot*	1.03	1.29	1.16
Left Forearm*	1.57	1.88	1.67
Left Hand*	0.48	0.70	0.60
Left Shank*	4.27	5.62	4.77
Left Thigh	13.57	14.48	N/A
Right Arm	3.45	3.79	N/A
Right Foot*	1.03	1.30	1.13
Right Forearm*	1.47	1.65	1.53
Right Hand*	0.51	0.75	0.63
Right Shank*	3.98	5.21	4.42
Right Thigh	13.66	14.54	N/A
Torso	50.05	53.63	N/A
Total Body	103.99	114.27	111.78

Table S15: Segment mass (as % of body mass) of the original surface scan, convex hull, regression model and average values. S: Original detailed surface mesh. CH: Convex Hull of whole body segments. CHD: Convex Hull with subdivided body segments (only segments indicated with an * were subdivided as shown in main manuscript in Fig. 10). ZR: Values predicted using Zatsiosrky's linear regression model (using weight and height). Z: Male average values reported by Zatsiorsky. D: Male average values reported by Dempster .

Segment	S	CH	CHD	ZR (m)	Z (m)	D (m)
Foot*	1.02	1.17	1.05	1.30	1.37	1.43
Shank*	4.09	4.88	4.23	4.26	4.33	4.62
Thigh	13.48	13.06	13.36	14.40	14.17	10.00
Hand*	0.49	0.65	0.57	0.58	0.61	0.61
Forearm*	1.51	1.59	1.47	1.57	1.63	1.53
Upper Arm	3.29	3.23	3.30	2.75	2.70	2.64
Head and Neck	5.67	5.45	5.58	5.99	6.94	7.92
Torso	46.58	45.39	46.44	44.29	43.47	51.29

Table S16: Shift of the centre of mass (CoM) between original scan and hulled segments in [mm]. CH_x, CH_y, CH_z: Shift of CoM of the convex hulled segments with respect to the original scan. CHD_x, CHD_y, CHD_z: Shift of th CoM of the dived body segments (only segments indicated with an * were subdivided). Highlighted are values with a shift of larger than 5mm.

Segment	CH_x	CH_y	CH_z	CHD_x	CHD_y	CHD_z
Head	0.40	1.32	4.32	N/A	N/A	N/A
Left Arm	0.21	-1.84	2.39	N/A	N/A	N/A
Left Foot*	0.55	6.29	2.04	-0.42	2.50	0.65
Left Forearm*	5.52	5.15	2.52	0.18	0.19	0.24
Left Hand*	10.40	-1.43	9.15	5.63	0.15	6.91
Left Shank*	-0.26	7.80	14.81	0.04	2.55	4.17
Left Thigh	0.66	-2.52	3.68	N/A	N/A	N/A
Right Arm	-0.72	-0.14	4.76	N/A	N/A	N/A
Right Foot*	-1.65	5.34	2.12	0.05	1.99	0.84
Right Forearm*	-3.74	3.55	1.31	-0.11	0.10	0.10
Right Hand*	-10.55	-4.01	4.85	-5.35	-0.92	3.73
Right Shank*	-1.59	6.88	13.23	-0.66	2.57	3.10
Right Thigh	-0.44	-2.41	4.10	N/A	N/A	N/A
Torso	1.94	-1.97	1.65	N/A	N/A	N/A

Table S17: Inertial tensor values Visible Human high-resolution mesh (see Fig.8A in main manuscript) in [10^4 kg*m²]. Products of inertia sign convention such that they appear as -Ixy, -Ixz and -Iyz in the inertia matrix. Note: The visible human body segments were not rotated into the standard anatomical position, thus Ixx, Iyy and Izz do not correspond to principal body axes (see coordinate system in main manuscript Fig. 8).

**Body Segment:**	**Ixx**	**Iyy**	**Izz**	**Ixy**	**Ixz**	**Iyz**
Head	329.7	283.4	197.3	-0.6	9.3	1.7
Left Arm	224.4	204.4	58.9	3.1	-21.2	-15.8
Left Foot	33.6	14.3	27.2	-1.0	-0.7	10.5
Left Forearm	72.3	53.8	39.9	9.5	12.4	26.8
Left Hand	5.9	8.2	5.2	1.2	1.7	0.7
Left Shank	479.6	479.6	80.4	-1.6	-46.3	-18.8
Left Thigh	2226.3	2138.9	611.2	-13.4	158.0	53.4
Right Arm	283.2	260.4	61.6	-3.1	18.9	-7.5
Right Foot	33.2	16.8	23.7	-0.7	-0.1	11.1
Right Forearm	58.8	46.4	43.9	-14.3	-16.0	22.2
Right Hand	5.4	8.5	6.1	-1.0	-1.5	0.0
Right Shank	444.1	441.0	72.5	-2.1	47.2	-26.2
Right Thigh	2259.8	2155.2	619.2	-4.2	-124.9	62.0
Torso	16660.7	17909.0	5617.5	-53.4	-215.1	400.5

Table S18: Inertial tensor values Visible Human convex hull mesh (see Fig.8B in main manuscript) in [10^4 kg*m²]. Products of inertia sign convention such that they appear as -Ixy, -Ixz and -Iyz in the inertia matrix. Note: The visible human body segments were not rotated into the standard anatomical position, thus Ixx, Iyy and Izz do not correspond to principal body axes (see coordinate system in main manuscript Fig. 8).

**Body Segment:**	**Ixx**	**Iyy**	**Izz**	**Ixy**	**Ixz**	**Iyz**
Head	326.0	277.2	200.1	-1.4	9.1	3.3
Left Arm	219.1	196.9	60.0	2.0	-19.2	-17.0
Left Foot	39.2	16.6	32.9	-1.4	-0.8	11.4
Left Forearm	78.4	60.0	46.4	11.8	14.1	28.6
Left Hand	8.1	12.0	7.5	1.3	3.0	0.6
Left Shank	613.1	600.6	112.6	-3.2	-55.1	-19.3
Left Thigh	2193.3	2098.2	625.2	-16.8	156.1	28.2
Right Arm	286.9	257.6	68.0	-2.6	16.2	-6.5
Right Foot	38.5	19.3	28.9	-0.6	0.0	12.0
Right Forearm	59.3	47.6	46.3	-15.1	-16.1	21.8
Right Hand	7.0	11.8	9.0	-0.6	-2.2	-0.4
Right Shank	556.0	540.8	100.1	-3.3	53.6	-29.9
Right Thigh	2214.7	2106.1	628.6	-3.0	-121.3	40.2
Torso	16146.1	17431.3	5828.5	-45.2	-178.5	282.4

Table S19: Inertial tensor values Visible Human convex hull mesh of divided segments (see Fig.10 in main manuscript,only segments indicated with an * were subdivided) in [10^4 kg*m^2]. Products of inertia sign convention such that they appear as -Ixy, -Ixz and -Iyz in the inertia matrix. Note: The visible human body segments were not rotated into the standard anatomical position, thus Ixx, Iyy and Izz do not correspond to principal body axes (see coordinate system in main manuscript Fig. 8).

Body Segment:	Ixx	Iyy	Izz	Ixy	Ixz	Iyz
Head	333.3	283.4	204.5	1.5	-9.3	-3.4
Left Arm	224.0	201.3	61.4	-2.0	19.6	17.4
Left Foot*	36.0	15.3	29.2	0.9	0.7	-2.8
Left Forearm*	74.4	56.2	41.6	-10.5	-13.6	-27.8
Left Hand*	7.5	10.6	6.3	-1.3	-2.6	-0.8
Left Shank*	529.8	528.0	89.8	1.3	52.0	22.9
Left Thigh	2242.2	2145.0	639.1	17.2	-159.6	-28.9
Right Arm	293.3	263.3	69.5	2.6	-16.6	6.6
Right Foot*	34.6	17.5	25.0	0.6	0.0	-11.6
Right Forearm*	57.8	45.9	43.5	14.3	15.8	-21.8
Right Hand*	6.3	10.2	7.3	0.8	2.0	0.1
Right Shank*	486.9	481.2	80.6	2.7	-51.8	31.4
Right Thigh	2264.1	2153.1	642.6	3.1	3.1	-41.1
Torso	16506.3	17820.2	5958.6	46.2	182.5	-288.7

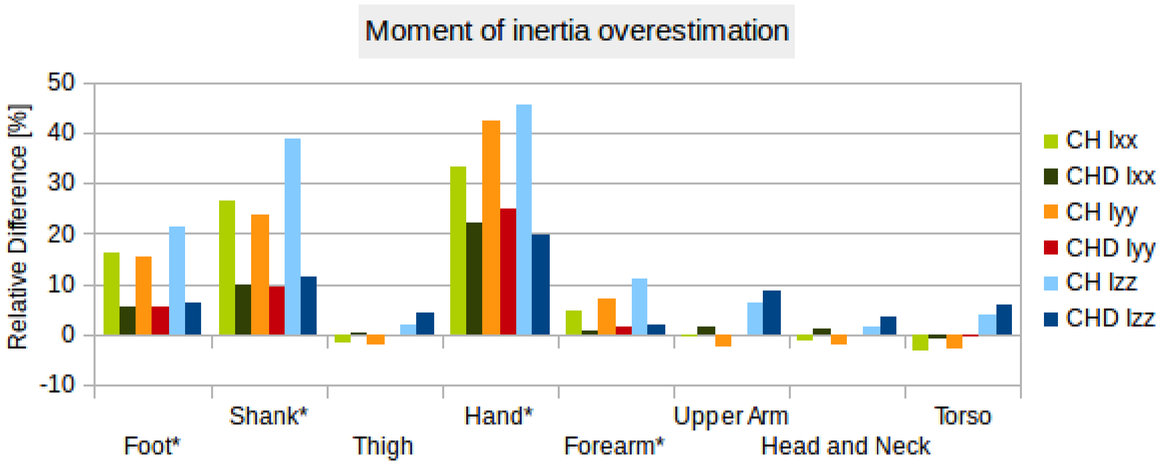

Figure S2: Moment of inertia overestimation of the hulled versus original mehs of the visible human data set CH: Convex hull, CHD: Divided Convex Hull. Note: The visible human body segments were not rotated into the standard anatomical position, thus Ixx, Iyy and Izz do not correspond to principal body axes (see coordinate system in main manuscript Fig. 8).
